# Shape Sensing in Plate Structures through Inverse Finite Element Method Enhanced by Multi-Objective Genetic Optimization of Sensor Placement and Strain Pre-Extrapolation

**DOI:** 10.3390/s24020608

**Published:** 2024-01-18

**Authors:** Emiliano Del Priore, Luca Lampani

**Affiliations:** Dipartimento di Ingegneria Meccanica e Aerospaziale, Sapienza Università di Roma, 00184 Rome, Italy; emiliano.delpriore@uniroma1.it

**Keywords:** structural health monitoring, shape sensing, sensor placement, inverse finite element method, smoothing element analysis, multi-objective genetic algorithm

## Abstract

The real-time reconstruction of the displacement field of a structure from a network of in situ strain sensors is commonly referred to as “shape sensing”. The inverse finite element method (iFEM) stands out as a highly effective and promising approach to perform this task. In the current investigation, this technique is employed to monitor different plate structures experiencing flexural and torsional deformation fields. In order to reduce the number of installed sensors and obtain more accurate results, the iFEM is applied in synergy with smoothing element analysis (SEA), which allows the pre-extrapolation of the strain field over the entire structure from a limited number of measurement points. For the SEA extrapolation to be effective for a multitude of load cases, it is necessary to position the strain sensors appropriately. In this study, an innovative sensor placement strategy that relies on a multi-objective genetic algorithm (NSGA-II) is proposed. This approach aims to minimize the root mean square error of the pre-extrapolated strain field across a set of mode shapes for the examined plate structures. The optimized strain reconstruction is subsequently utilized as input for the iFEM technique. Comparisons are drawn between the displacement field reconstructions obtained using the proposed methodology and the conventional iFEM. In order to validate such methodology, two different numerical case studies, one involving a rectangular cantilevered plate and the other encompassing a square plate clamped at the edges, are investigated. For the considered case studies, the results obtained by the proposed approach reveal a significant improvement in the monitoring capabilities over the basic iFEM algorithm with the same number of sensors.

## 1. Introduction

A structural health monitoring (SHM) system integrates strategically placed sensors to gather essential data about the structure and its environment, enabling a comprehensive assessment of its overall condition [1]. The primary aim is not only to evaluate the structural integrity, but also to monitor and check the system’s performance in operational scenarios [2]. The combination of SHM and non-destructive testing offers detailed insight into the structural conditions, optimizing maintenance strategies and ensuring long-term safety [3]. Shape sensing is a specific branch of SHM dedicated to the real-time reconstruction of the displacement field of a structure from a set of in situ strain measurements. It allows for the continuous tracking of both static and dynamic responses within the system [4]. Furthermore, reconstructing the displacement field serves as an initial step in assessing stresses across either the entire structure or specific regions [5]. The attention towards shape-sensing techniques has increased alongside the advent of new strain sensor technologies. In addition to traditional strain gauges, contemporary solutions incorporate fiber optic sensors (FOSs). FOSs offer notable advantages, such as insensitivity to electromagnetic radiation, light weight, small size, great sensitivity and resolution, and suitability to be embedded into structures [6] These sensors have found numerous shape-sensing applications in civil and aerospace engineering [7].

The methods for solving shape-sensing problems can be broadly classified into four categories [8]. The first category encompasses analytical approaches, such as Ko’s displacement theory [9], which involves direct or numerical integration of experimentally measured strain. The second category includes methods that utilize continuous basis functions to approximate displacements. Modal methods (MM) [10,11], for example, fall into this class as they use known spatial functions like mode shapes to describe the displacement field, with unknown weights determined through a curve-fitting process using experimental strains. The third category involves computational models that use artificial neural networks (ANNs) [12]. The fourth, and last, class is represented by the inverse finite element method (iFEM), developed by Tessler and Spangler [13]. This technique is based on the least-squares variational principle and finite element discretization. Ko’s displacement theory is limited to beam-like structures as it relies on the kinematic assumptions in the Euler–Bernoulli theory. On the other hand, methods employing ANNs have not been applied widely due to their results being heavily reliant on training datasets. Thus, among the four classes, MM and iFEM exhibit higher general applicability to various kinds of structures. Few studies in the literature focus on comparing these two approaches. For instance, ref. [14] provides an experimental comparison for a stiffened panel equipped with fiber optic sensors, while ref. [15] presents numerical analysis involving iFEM, MM, and Ko’s theory for a composite wingbox. These works emphasize that MM is a useful tool in the presence of uncertainties, since it is less influenced by them, although it achieves a lower reconstruction accuracy than iFEM. Other than its high accuracy, the inverse finite element method’s key strengths lie in its ability to monitor the displacement field even under unknown loading conditions and material properties [16], along with its computational speed which enables real-time applications.

Over the past decade, a variety of inverse elements has been developed on the basis of the first-order shear deformation theory (FSDT). Among the FSDT-based iFEM elements is a three-node inverse-shell element, designated as iMIN3 [17]; a four-node quadrilateral inverse-shell element, denoted as iQS4 [18]; an eight-node curved inverse shell element, referred to as iCS8 [19]; and a two-node inverse-beam element [20]. In addition, Kefal and Oterkus [21] have introduced a novel isogeometric inverse element (iKLS), which utilizes Non-Uniform Rational B-Splines (NURBS) as shape functions to achieve a geometrically exact representation of curved thin shell structures. Subsequently, this isogeometric approach has also been extended to the reconstruction of the displacement field in beam-like geometries [22,23]. The abovementioned elements enable real-time monitoring of isotropic structures like beams, plates, and shells by means of the inverse finite element method. More recently, a novel class of elements [24] has been introduced, leveraging the refined zig-zag theory [25], to exploit the iFEM technique in moderately thick sandwich and laminated composite structures. Over the last few years, iFEM has found both numerical and/or experimental applications in shape-sensing analyses across various interesting case studies, including marine vessels [26], offshore wind turbine towers [27], and stiffened panels [14,28,29].

The precision of the inverse finite element method in reconstructing the displacement field is significantly influenced by the number of sensors used. The installation of hundreds of sensors, required to achieve a low error, may be prohibitively expensive and is to be avoided in particular industries, like aerospace, where weight is an important factor to be taken into account. Thus, it may be useful to make use of pre-extrapolation techniques to obtain the input strain field on a more refined mesh from a reduced number of sensors. Notable works addressing this issue include those by Abdollahzdeh et al. [30] and Kefal et al. [31]. The first study employed polynomial extrapolation to approximate the strain field from sensor data, while the latter utilized a smoothing procedure based on the minimization of penalized least-squares functionals, known as smoothing element analysis (SEA) [32]. SEA discretizes the structural domain using least-squares finite elements, where the variable to be smoothed (e.g., stress or strain) is interpolated within each element using piecewise continuous functions. Oboe et al. [33] conducted a comparison between these two pre-extrapolation techniques on a plate subjected to a compressive load. The authors observed that SEA demonstrated a higher level of adaptability to the input strain field, allowing it to achieve more versatile results.

The accuracy of pre-extrapolation performed with SEA depends on the sensor placement and on the complexity of the strain field. For example, the uniform distribution of sensors on the structure may not optimally reconstruct the strain field since it might not correctly identify its peaks. Consequently, optimizing sensor placement becomes crucial to enhance the effectiveness of the pre-extrapolation process under specific loading conditions. Within the iFEM framework, various studies have focused on optimizing sensor placement. As an example, Zhao et al. [34] utilized particle swarm optimization (PSO) to derive optimal sensor schemes for beam structures based on eigenvalue analysis. Ghasemzadeh et al. [35] employed a genetic algorithm to strategically position sensors on selected inverse elements within the mesh of simple 2D and 3D structures under concentrated and distributed loads. Their approach aimed at minimizing the error in the reconstructed displacement field as the objective function. However, this study did not incorporate any pre-extrapolation mechanism, and each optimization was conducted based on a single predefined load (a sensor pattern was optimal only for a single loading condition). In contrast, Roy et al. [36] explored the reconstruction of two mode shapes of a cantilevered plate using iFEM pre-extrapolated with SEA. Although they considered efficient and easy-to-implement sensor placement patterns, these were not the result of an optimization process.

In this study, we achieve full-field displacement reconstruction using iFEM with pre-extrapolated SEA strains as input. To enhance the effectiveness of pre-extrapolation, we introduce an innovative approach employing a genetic algorithm to optimize sensor placement, aiming to closely match the pre-extrapolated strain field with the actual one. Ensuring the validity of the sensor pattern across diverse loading conditions, the optimization process is multi-objective, utilizing the well-established non-dominated sorting genetic algorithm (NSGA-II [37]). Specifically, we focus on analyzing a cantilevered rectangular plate and a square plate clamped on all four sides, optimizing sensor placement for the improved reconstruction of a pre-selected set of vibration mode shapes. A comparative analysis is conducted between the displacement field reconstructions obtained through our proposed methodology, the conventional iFEM, and the combined use of the iFEM and SEA with a uniformly distributed sensor pattern. The results from our approach reveal a significant enhancement in monitoring capabilities compared to the standard iFEM algorithm, despite utilizing the same number of sensors.

## 2. Theoretical Background

### 2.1. Inverse Finite Element Method

Within the iFEM framework, the structural domain is discretized with a set of inverse finite elements. The algorithm computes the nodal displacements of the discretized mesh, taking into account the structure’s geometry, boundary conditions, and a set of in situ strain measurements.

In the present work, an element formulation based on the first-order shear deformation theory has been adopted. Therefore, the displacement field can be described in terms of the membrane displacements, $u\left(x\right)$ and $v\left(x\right)$ , the deflection $w\left(x\right)$ , as well as the rotations around the mid-plane axis x and y , expressed by $\theta_{x}\left(x\right)$ and $\;\theta_{y}\left(x\right)$ , respectively ( x denotes the mid-plane coordinates). The Cartesian displacement components are written as:

(1) $$u_{x}=u\left(x\right)+z\theta_{y}\left(x\right)\;u_{y}=v\left(x\right)-z\theta_{x}\left(x\right)\;u_{z}=w\left(x\right)$$

where $z\in \left[-h,+h\right]$ indicates the through-the-thickness coordinate.

The strain field is obtained by derivation of the kinematic variables:

(2) $$\left\{\begin{array}{c} \varepsilon_{xx}\\ \varepsilon_{yy}\\ \gamma_{xy} \end{array}\right\}=\left\{\begin{array}{c} u_{x,x}\\ u_{y,y}\\ u_{x,y}+u_{y,x} \end{array}\right\}=\left\{\begin{array}{c} u_{,x}\\ v_{,y}\\ v_{,x}+u_{,y} \end{array}\right\}+z\left\{\begin{array}{c} \theta_{y,x}\\ -\theta_{x,y}\\ \theta_{y,y}-\theta_{x,x} \end{array}\right\}=e+zk\;\left\{\begin{array}{c} \gamma_{xz}\\ \gamma_{yz} \end{array}\right\}=\left\{\begin{array}{c} u_{x,z}+u_{z,x}\\ u_{y,z}+u_{z,y} \end{array}\right\}=\left\{\begin{array}{c} w_{,x}+\theta_{y}\\ w_{,y}-\theta_{x} \end{array}\right\}=g$$

where e , k , and g are the membrane, bending, and transverse shear section strains, respectively.

**Figure 1:**
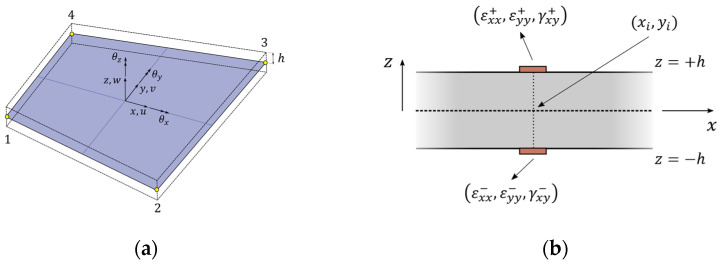
(**a**) iQS4 element; (**b**) discrete surface strains at i-th measurement point.

The goal of the inverse finite element method is to determine the nodal degrees of freedom values that minimize the error between the reconstructed strain field and the one measured using a discrete set of points. For a single inverse element, the error in the reconstruction is quantified by means of a weighted least-squares functional, defined as follows:

(3) $$\Phi^{e}=w_{m}{\left\|\left(e_{i}-e_{i}^{\varepsilon }\right)\right\|}^{2}+w_{b}{\left(2h\right)}^{2}{\left\|\left(k_{i}-k_{i}^{\varepsilon }\right)\right\|}^{2}+w_{s}{\left\|\left(g_{i}-g_{i}^{\varepsilon }\right)\right\|}^{2}$$

where the superscript ε is used to denote the values measured from an in situ strain sensor. In Equation (3) $w_{m}$ , $w_{b}$ , and $w_{s}$ are positive-valued weighting constants associated with membrane strains, bending curvatures, and shear strains, respectively. In the present work, the structure’s geometry is discretized with four-node inverse elements, known in the literature as iQS4 (displayed in Figure 1a). The kinematic variables inside each element are, thus, interpolated using a set of anisoparametric [38] shape functions:

(4) $${\left[u\;\;v\;\;w\;\;\theta_{x}\;\theta_{y}\right]}^{T}=N^{e}u^{e}\;\;\;\;\;\;\;\;\left(\;u^{eT}=\left[u_{i}\;\;v_{i}\;\;w_{i}\;\;\theta_{xi}\;\theta_{yi}\;\theta_{zi}\right],\;i=1,\;\ldots ,\;4\right)$$

In this context, $N^{e}$ denotes the matrix of the shape functions, and $u^{e}$ is the vector representing the nodal degrees of freedom for each element. A single element is characterized by 24 DOF, consisting of three displacements and three rotations per node. It should be noted that $\theta_{zi}$ denotes the drilling rotation of the i-th node. Although not considered a kinematic variable in the FSDT formulation, it is utilized in the element interpolation of membrane displacements. By substituting Equation (4) into Equation (2), the numerical strain components can be expressed as the product between the matrices $B_{m}$ , $B_{b}$ , and $B_{s}$ containing the derivatives of the shape functions, and the element nodal displacements $u^{e}$ .

(5) $$e\left(u^{e}\right)=B_{m}u^{e}\;\;\;\;\;\;\;k\left(u^{e}\right)=B_{b}u^{e}\;\;\;\;\;\;\;g\left(u^{e}\right)=B_{s}u^{e}$$

If an element is equipped with n strain sensors, the least-squares functional can be rewritten by means of the Euclidean norm, as follows:

(6) $$\Phi^{e}\left(u^{e}\right)=\frac{1}{n}\left(w_{m}\int_{A_{e}}\overset{n}{\underset{i=1}{\sum }}{\left(e{\left(u^{e}\right)}_{i}-e_{i}^{\varepsilon }\right)}^{2}+w_{b}{\left(2h\right)}^{2}\int_{A_{e}}\overset{n}{\underset{i=1}{\sum }}{\left(k{\left(u^{e}\right)}_{i}-k_{i}^{\varepsilon }\right)}^{2}+w_{s}\int_{A_{e}}\overset{n}{\underset{i=1}{\sum }}{\left(g{\left(u^{e}\right)}_{i}-g_{i}^{\varepsilon }\right)}^{2}\right)$$

By inserting Equation (5) into the functional of Equation (6) and minimizing it with respect to $u^{e}$ , one obtains a system of linear equations:

(7) $$\frac{\partial \Phi \left(u^{e}\right)}{\partial u^{e}}=0\;\;\;\;\to \;\;\;\;K^{e}u^{e}=F^{e}$$

with

(8) $$K^{e}=\int_{A_{e}}\left(w_{m}{B^{m}}^{T}B^{m}+{\left(2h\right)}^{2}{B^{b}}^{T}B^{b}+w_{s}{B^{s}}^{T}B^{s}\right)dA$$

(9) $$F^{e}=\frac{1}{n}\int_{A_{e}}\overset{n}{\underset{i=1}{\sum }}\left(w_{m}{B^{m}}^{T}e_{i}^{\varepsilon }+{\left(2h\right)}^{2}{B^{b}}^{T}k_{i}^{\varepsilon }+w_{s}{B^{s}}^{T}g_{i}^{\varepsilon }\right)dA$$

Given a structure equipped with a set of surface-mounted strain sensors, the experimental membrane and curvature strains at the i-th measurement point (Figure 1b) can be derived as follows:

(10) $$e_{i}^{\varepsilon }=\frac{1}{2}\left\{\begin{array}{c} \varepsilon_{xx}^{+}+\varepsilon_{xx}^{-}\\ \varepsilon_{yy}^{+}+\varepsilon_{yy}^{-}\\ \varepsilon_{xy}^{+}+\varepsilon_{xy}^{-} \end{array}\right\}\;\;\;\;\;\;\;\;k_{i}^{\varepsilon }=\frac{1}{2h}\left\{\begin{array}{c} \varepsilon_{xx}^{+}-\varepsilon_{xx}^{-}\\ \varepsilon_{yy}^{+}-\varepsilon_{yy}^{-}\\ \varepsilon_{xy}^{+}-\varepsilon_{xy}^{-} \end{array}\right\}$$

The superscripts + and − refer to the quantities corresponding to the top and bottom surface, respectively. It is not mandatory to equip every element in the mesh with sensors; the iFEM technique remains numerically stable even if certain elements lack in situ strain measurements. In such instances, the associated weights are assigned smaller values, e.g., $10^{-4}$ (otherwise they are set to unity). In general, transverse shear strains cannot be directly measured using surface-mounted strain gauges: therefore, $g_{i}^{\varepsilon }$ is considered as 0 , and the corresponding weight, $w_{s}$ , is selected to be small.

The matrices $K^{e}$ and $F^{e}$ , computed for each element within the domain, can be assembled into a system of linear equations for the global degrees of freedom:

(11) KU=F

where K is a matrix depending on the shape functions and strain-sensor locations, whereas F is a vector which encompasses the measured data. The matrix K is computed and inverted only once (after applying the appropriate boundary conditions) in the monitoring process, while the vector F needs to be updated each time new strain measures are acquired.

### 2.2. Smoothing Element Analysis

To enhance the precision and cost effectiveness of shape-sensing analysis, especially when dealing with a limited number of sensors, a viable strategy involves pre-extrapolating the structure’s strain field. The inverse problem is then addressed by means of the iFEM, utilizing the pre-extrapolated data as an input [39]. This approach enables the use of a finer mesh with a large number of measurement samples, ensuring higher accuracy in the reconstruction of the displacement field.

**Figure 2:**
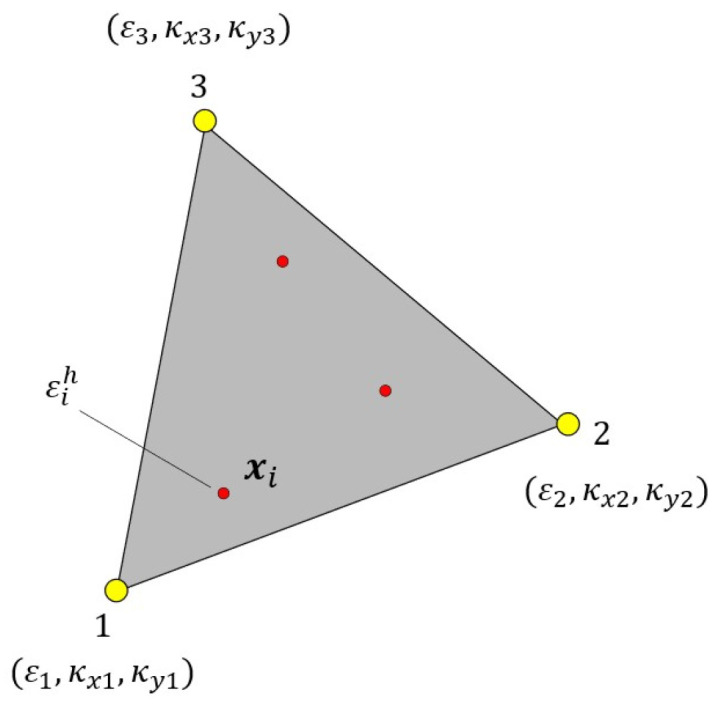
Triangular smoothing element with nodes 1, 2, 3 and their corresponding DOF.

Smoothing element analysis (SEA) is a robust technique, which can be employed to pre-extrapolate the input strain field. The numerical formulation of SEA relies on a variational principle employing a penalized discrete least-squares (PDLS) functional [40]. The algorithm adopts a finite element approach, in which the geometry is discretized into a triangular mesh. Let us define a general measured strain as $\varepsilon_{i}^{h}$ (which could be $\varepsilon_{xx}^{+}$ , for example), while the corresponding strain after smoothing is denoted as $\varepsilon \left(x_{i}\right)$ . For a single mesh element (shown in Figure 2), the PDLS functional is expressed as:

(12) $$\Psi =\frac{1}{n}\overset{n}{\underset{i=1}{\sum }}{\left.\left(\;\varepsilon \left(x_{i}\right)-\varepsilon_{i}^{h}\right.\right)}^{2}+\alpha \int_{S}\left.\left(\;{\left.\left(\;\varepsilon_{,x}-\kappa_{x}\right.\right)}^{2}+{\left.\left(\;\varepsilon_{,y}-\kappa_{y}\right.\right)}^{2}\right.\right)dS+\beta S\;\int_{S}\left.\left(\;{\left.\left(\;\kappa_{x,x}\right.\right)}^{2}+{\left.\left(\;\kappa_{y,y}\right.\right)}^{2}+\frac{1}{2}{\left.\left(\;\kappa_{x,y}+\kappa_{y,x}\right.\right)}^{2}\right.\right)dS$$

Here, n is the total number of measurements within the element; ${\left(\cdot \right)}_{,x}$ and ${\left(\cdot \right)}_{,y}$ represent the partial derivative operator with respect to x and y ; $\kappa_{x}$ and $\kappa_{y}\;$ are the analytical counterparts of the partial derivatives of the experimental strain along directions x and y , respectively. The first term in the equation is a discrete least-squares functional, which enforces a match between the extrapolated strain field and the experimental strain data. The second term introduces a penalty constraint functional, which depends on the dimensionless parameter α . For large values of α (e.g., $10^{2}–10^{6}$ ), such term ensures the limiting condition of $C^{1}$ continuity for ε . The third term serves as a regularization functional, relying on the positive parameter β . This term imposes an additional constraint on the derivatives $\kappa_{1}$ and $\kappa_{2}$ , the severity of which is governed by the value of the parameter β . Particularly, when the sampled data is reasonably accurate, β should be significatively small with respect to α (e.g., $10^{-3}–10^{-4}$ ).

The field variables are expressed with an anisoparametric interpolation within each element:

(13) $$\varepsilon =Ps+Ms_{x}+Ls_{y}\;\kappa_{x}=Ps_{x}\;\kappa_{y}=Ps_{y}$$

where $s={\left[\varepsilon_{1},\;\varepsilon_{2},\;\varepsilon_{3}\right]}^{T}$ , $\;\;s_{x}={\left[\kappa_{x1},\;\kappa_{x2},\;\kappa_{x3}\right]}^{T}$ , $s_{y}={\left[\kappa_{y1},\;\kappa_{y2},\;\kappa_{y3}\right]}^{T}$ are the vectors of the nodal DOF of the element, P is a row vector of linear shape functions, while M and L are the row vectors of quadratic shape functions. We can define $d^{e}={\left[s,\;s_{x},s_{y}\right]}^{T}$ , an array containing the nine unknowns of each element. By substituting Equation (13) into the functional of Equation (12), the condition for which Ψ is minimized can be written as:

(14) $$\frac{\partial \Psi \left(d^{e}\right)}{\partial d^{e}}=0\;\Rightarrow A^{e}d^{e}=H^{e}$$

(15) $$A^{e}=\frac{1}{n}\overset{n}{\underset{i=1}{\sum }}\left.\left[\;{\left(N\left(x_{i}\right)\right)}^{T}\left(N\left(x_{i}\right)\right)\right.\right]+\alpha \;\int_{s}{Z_{\alpha }}^{T}Z_{\alpha }\;dA+\beta S\;\int_{s}{Z_{\beta }}^{T}D_{\beta }Z_{\beta }\;dA$$

(16) $$H^{e}=\frac{1}{n}\;\overset{n}{\underset{i=1}{\sum }}{\left(N\left(x_{i}\right)\right)}^{T}\varepsilon_{i}^{h}$$

In which:

(17) $$N={\left[P,\;M,\;L\right]}^{T}$$

(18) $$Z_{\alpha }=\left[\begin{array}{ccc} P_{,x} & M_{,x}-P & L_{,x}\\ P_{,y} & M_{,y} & L_{,y}-P \end{array}\right]$$

(19) $$Z_{\beta }=\left[\begin{array}{ccc} 0 & P_{,x} & 0\\ 0 & 0 & P_{,y}\\ 0 & P_{,y} & P_{,x} \end{array}\right]$$

(20) $$D_{\beta }=\left[\begin{array}{ccc} 1 & 0 & 0\\ 0 & 1 & 0\\ 0 & 0 & 1/2 \end{array}\right]$$

Note, that if an element does not have any input strain, both $H^{e}$ and the first term of $A^{e}$ will be absent, and the second and third term of Equation (15) will be the only ones contributing to the element stiffness matrix.

After discretizing the geometry with multiple smoothing elements, a global system of equations is assembled, which can easily be solved for the unknown nodal degrees of freedom of the mesh (i.e., the strain field, and its derivatives in each node). Although the imposition of boundary conditions is not mandatory in smoothing element analysis, they may be applied if the exact solution is known in specific regions [33]. Once the linear system is solved, it is possible to compute the strain field at any arbitrary point in the domain by means of the shape functions.

### 2.3. Multi-Objective Genetic Algorithm

A genetic algorithm (GA) is a heuristic optimization approach inspired by natural selection principles [41]. The process involves initiating a population, conducting reproduction, mutating genes, and applying natural selection to attain an optimal population. In a GA, a chromosome (or individual) represents a potential solution to the problem the algorithm is trying to solve. Chromosomes are composed of discrete units known as genes. Each gene regulates specific features of the chromosome. A GA operates with a collection of chromosomes, forming a population that is typically initialized randomly.

As the search progresses, the population evolves to include increasingly fit solutions, until convergence is reached. A GA employs two key operators for generating new solutions from existing ones: crossover and mutation [42]. In the crossover process, two chromosomes, known as parents, are combined to produce an offspring. The parents are chosen using a selection operator that favors fitter individuals from the existing population. Through the iterative application of the crossover operator, genes from superior chromosomes are more likely to be incorporated into the population, contributing to the overall convergence to a favorable solution. The mutation operator introduces random changes to the characteristics of the chromosomes. Mutation is pivotal in a GA as it injects genetic diversity into the population, aiding the search in escaping local optima. By combining these mechanisms, a GA dynamically refines its population, iteratively improving solutions to reach an optimal outcome.

For multi-objective optimization problems, attaining a solution that is optimal for every objective is often impossible [43]. Instead, the concept of Pareto domination and Pareto optimal solutions are introduced. NSGA-II, a multi-objective genetic algorithm, serves as an optimization tool to find a Pareto-optimal set of solutions. NSGA-II works similarly to a single-objective GA, but incorporates specific operations in the selection process [44]:

1. Fast non-dominated sorting approach: The population is sorted into different non-dominated fronts. Individuals in the first non-dominated front are identified, and their rank is set to 1. The remaining individuals in the population, excluding those with rank 1, continue to be sorted using the same procedure. This process continues until all fronts are identified.
2. Crowding distance assignment: Individuals with the same rank are arranged based on the crowding distance, which represents the average distance between a solution and its neighboring solutions on the front in the objective space.
3. Selection operator: The binary tournament selection is employed to choose the parents. Two individuals are randomly selected from the population, and if their ranks differ, the one with the smaller rank is chosen. If their ranks are the same, the individual with the larger crowding distance is selected. This method ensures a more even distribution of solutions along the Pareto front, preventing overcrowding in specific regions.

### 2.4. Proposed Methodology

In this study, SEA is employed to pre-extrapolate the strain field, which serves as input for the inverse finite element method. Through the smoothing process, a continuous description of the strain field is produced, allowing the application of the iFEM on a fine mesh. The use of a fine mesh is theoretically feasible even with a relatively low number of sensors in the standard iFEM, as the weights of the individual elements can be adjusted to account for the presence or absence of a measurement. However, maintaining a sufficient ratio between the number of elements with strain data and the total number of elements in the mesh is crucial to avoid a significant degradation of the results. Therefore, employing SEA allows the use of particularly dense meshes, where each element has measurement data, ensuring high accuracy. To obtain good results though, it is crucial that the pre-extrapolated strain field closely approximates the real one. To accomplish this, we optimize the sensor placement process by means of a genetic algorithm. Since a structure can undergo various loading conditions, it makes sense to perform a multi-objective optimization based on multiple operational scenarios. Therefore, the proposed optimization strategy involves the use of NSGA-II. For the case studies under consideration, we optimized the sensor placement to enable SEA to accurately reconstruct the deformation fields associated with a specific set of structural mode shapes. We made this choice since it is generally possible to decompose a general linear state of deformation into a sum of weighted mode shapes.

The starting point in the process is the finite element (FE) model of the structure (in our case a plate), which serves a dual purpose: it generates the discrete strain data (simulating what would be read by actual sensors in real-world applications) and provides a reference for evaluating the accuracy of the results obtained from the shape-sensing code. A modal analysis is performed on the FE model, allowing us to extract the first $N_{m}$ natural modes of the structure. The number of sensors to be placed on the structure, denoted as $N_{s}$ , is determined a priori. It is chosen in such a way that the measurement points are sufficient for pre-extrapolating the strain field in an appropriate manner, considering both the field behavior and the error level; consequently, a more complex strain field requires a higher number of installed sensors. Certainly, the number of measurement points was limited to a reasonable value, ensuring that the use of pre-extrapolation remains beneficial. Therefore, the goal of the optimization is to find a set of Pareto optimal sensor positions. The possible locations for the sensors are selected among the centroids of four-node quadrilateral shell elements in the FE mesh. Once the number of sensors to be installed is determined, a generic chromosome is defined by a matrix of $N_{s}$ rows. Each row (gene) is a vector $\left(x_{s},\;y_{s}\right)$ of the coordinates extracted from the set of centroids belonging to the FE mesh. The number of individuals in the population is referred to as $N_{pop}$ . The initial population is randomly generated, with care taken to avoid the rare event of duplicating the same individual in the process. In a subsequent step, the fitness of each chromosome is evaluated. Thus, for each chromosome, the six strain components $\left(e_{xx},\;e_{yy},\;e_{xy},\;k_{xx},\;k_{yy},\;k_{xy}\right)$ are reconstructed through smoothing element analysis, and the pre-extrapolated values are compared with the FEM ones at a set of sampling points for each target mode. In this work, since all the considered cases exhibit zero membrane strain, the objective function to minimize is chosen as the sum of the root mean square error in the SEA extrapolation across the three curvature components for a single mode:

(21) $$f_{r}=w_{r_{1}}\sqrt{\frac{1}{S}\overset{S}{\underset{i=1}{\sum }}{\left({k_{xx}}_{i}^{SEA}-{k_{xx}}_{i}^{FEM}\right)}^{2}}+w_{r_{2}}\sqrt{\frac{1}{S}\overset{S}{\underset{i=1}{\sum }}{\left({k_{yy}}_{i}^{SEA}-{k_{yy}}_{i}^{FEM}\right)}^{2}}+w_{r_{3}}\sqrt{\frac{1}{S}\overset{S}{\underset{i=1}{\sum }}{\left({k_{xy}}_{i}^{SEA}-{k_{xy}}_{i}^{FEM}\right)}^{2}}\;\;\;\;\;\;\;\;\;\;\left(r=1,\ldots ,\;N_{m}\right)$$

where S is the number of sampling points in which the rms error is evaluated and $w_{r_{i}}\;\left(i=1,\;2,\;3\right)$ are the weights associated with the rms error of each component. In our study, each weight was set to 1, although, in general, it is theoretically possible to make more suitable choices based on the deformation field to be pre-extrapolated. Since we are in a multi-objective optimization context, we will need to evaluate $N_{m}$ different fitness functions (one for each mode shape), with the goal of finding the Pareto optimal solutions.

**Figure 3:**
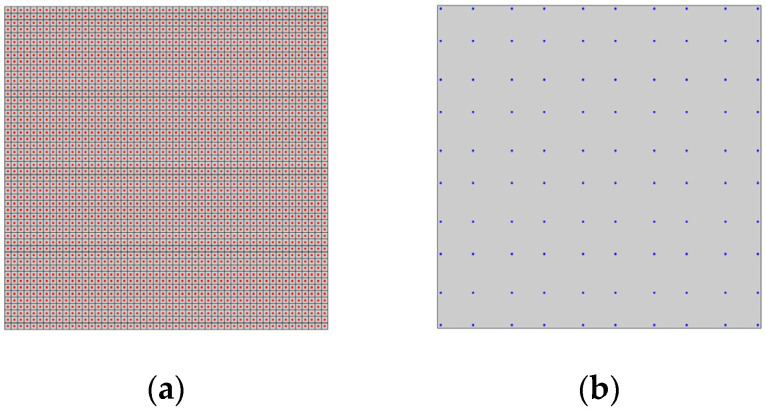
(**a**) Example of FE mesh with element centroids; (**b**) example of evaluation points.

In this study, the sampling points have been selected as a subset of the centroids of the FE mesh. In principle, the entire set of centroids could be used for this purpose, but given the large number, it leads to a significant increase in computational time when running the genetic algorithm. In Figure 3a, a square plate with the FE mesh and the set of centroids from which possible sensor coordinates are extracted is presented as an example, while in Figure 3b, a subset of sampling points is illustrated.

**Figure 4:**
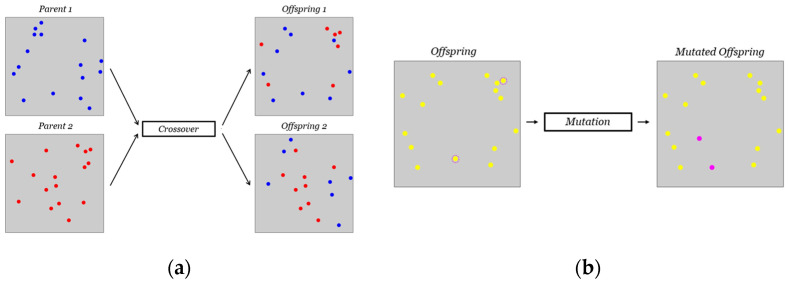
(**a**) Uniform crossover: blue dots represent sensor positions coming from Parent 1 meanwhile red dots are those coming from Parent 2; (**b**) mutation process: yellow dots represent sensor positions of the original offspring meanwhile purple dots are the mutated ones.

After evaluating the fitness of each individual in the population, a ranking is performed based on the Pareto fronts and crowding distance, as described in the previous section. To create the next generation, the parents are selected through binary tournament selection. Crossover can occur with a probability denoted as $p_{cross}$ . The present study employs a uniform crossover, in which each gene is selected randomly from one of the corresponding genes of the parent chromosomes with equal probability. In Figure 4a, an example of such crossover in our problem is illustrated. The blue and red dot patterns on the left represent Parent 1 and Parent 2, respectively. Following the crossover, the two resulting children inherit some sensor positions from Parent 1 (blue dots) and the remaining positions from Parent 2 (red dots). After the reproduction phase, some offspring may undergo a mutation process. For each offspring, a random real number between 0 and 1 is drawn, and if it exceeds $p_{mut}$ , the mutation probability, then the mutation occurs. In this work, mutation involves changing the positions of $n_{mut}$ random sensors belonging to the individual’s pattern with other random positions, among the set of centroids in the FE model. Moreover, $n_{m}$ , the number of mutated genes, varies between 1 and $n_{mut}^{max}$ (it should not be too high otherwise its effects may be too disruptive). In the studied cases, the value $n_{mut}^{max}$ varied depending on the number of sensors to be installed on the structure. Figure 4b shows an example of the mutation in our approach: on the left, the individual before mutation with two circled sensors randomly chosen to undergo the process; on the right, the individual after mutation, with the purple dots indicating the new positions of the two mutated sensors.

**Figure 5:**
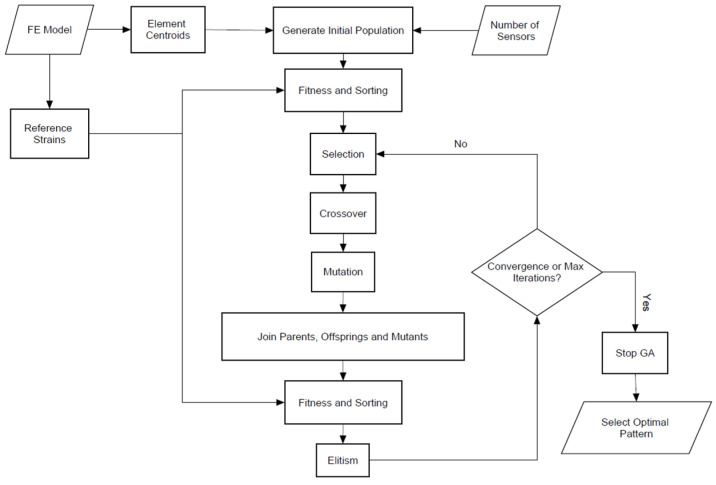
Flowchart of the optimization process.

At the end of the mutation process, elitism is applied to form the new population for reproduction, keeping only the best $N_{pop}$ individuals among the parents, offspring, and mutated individuals. A ranking is needed to choose the best $N_{pop}$ solutions and, thus, the non-dominated sorting and crowding distance are employed. The use of elitism ensures that there is no loss of good genes between generations, leading to a monotonic convergence into the optimal solution. The selection, crossover, mutation, and elitism operators are applied at each iteration of the genetic algorithm until convergence, or a maximum number of iterations is reached, at which point the whole process is terminated. In Figure 5, a flowchart that depicts the main steps in the optimization process is depicted.

## 3. Case Studies

The proposed approach is validated using two different case studies: a rectangular cantilevered plate and a clamped square plate.

### 3.1. Rectangular Cantilevered Plate

**Figure 6:**
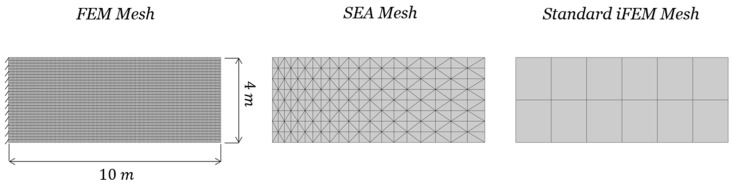
Cantilevered rectangular plate. FE mesh (**left**); SEA mesh (**center**); standard iFEM mesh (**right**).

The first case study focuses on a cantilevered plate, as depicted in Figure 6, with dimensions of 10 m in length, 4 m in width, and a thickness of 0.01 m . On the left vertical side, the following geometric constraints are enforced: u=v=w=0  and $\theta_{x}=\theta_{y}=\theta_{z}=0$ . The structure is assumed to be made of an isotropic material, with properties similar to an aluminum alloy ( E=70 Gpa , ν=0.3 , $\rho =2700\;\mathrm{kg}/m^{3}$ ). The plate was modeled using MSC NASTRAN, with a mesh comprising 162 × 30 CQUAD4 elements, which was selected following a convergence study, as illustrated on the left in Figure 6. Employing the SOL 103 solver, a modal analysis was conducted to extract the structure’s first three natural modes, identified at frequencies of 0.0841 Hz , 0.4398 Hz , and 0.5247 Hz . The first and third modes exhibit a purely flexural behavior, while the second mode is a torsional one. As previously mentioned, the pre-extrapolation produces a continuous strain field across the entire structure, enabling the use of a finer mesh in the reconstruction process. Therefore, the mesh shown on the left of Figure 6 is also employed by the smoothed iFEM. In this case study, we make use of 12 strain rosettes for the monitoring of the three mode shapes. Indeed, if the membrane strain is zero, as in this problem, it is sufficient to place the strain rosettes in only one area, among the top and bottom surface of Figure 1b. To optimize the sensor positioning, it is necessary to define a mesh for the smoothing element analysis (SEA). This triangular mesh consists of 320 elements, as shown in the middle of Figure 6. It should be noted that the mesh is finer near the plate’s constraint and gradually becomes sparser away from it. This design aims to capture steep strain gradients near the constraint, effectively. The mesh dimensions of the SEA were chosen following a series of tests to ensure that the pre-extrapolation technique adequately captured the shape and values of the strain field, while also taking into account the computational time of the pre-extrapolation (which affects both the time needed in the optimization process and the applicability of the method in real-time applications).

The third, and final, mesh utilized in this analysis is illustrated on the right in Figure 6 and corresponds to the standard iFEM (without pre-extrapolation and optimization). It comprises 6 × 2 inverse elements, each featuring a strain rosette at the centroid. Thus, the number of elements in the standard iFEM mesh was chosen to be equal to the number of sensors to be installed. This is because, when so few measurement points are present on the structure, using a finer mesh with some empty elements leads to the degradation of the results.

**Figure 7:**
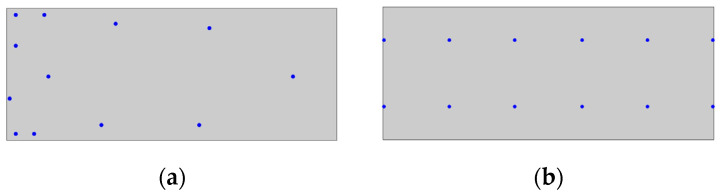
(**a**) Selected optimal sensor arrangement for the cantilevered rectangular plate; (**b**) uniform sensor placement used for validation.

The multi-objective genetic algorithm was run with a population of 300 individuals for 100 iterations, at the end of which convergence was achieved. The crossover and mutation probability are set at $p_{cross}=0.9$ and $p_{mut}=0.1$ , respectively; the maximum number of mutated genes is selected as $n_{mut}^{max}=2$ ; and the number of elites is equal to $N_{pop}$ (thus, selecting the top 300 individuals among the parents, offspring, and mutants). The rms error for the fitness function was calculated using a set of 280 sampling points taken from the centroids of the FEM mesh. The optimization process was repeated multiple times to account for the inherent randomness of the genetic algorithm. From all the runs, the final population that exhibited superior global fitness was selected for extracting a single solution, which was then used in the comparison with the standard iFEM. To pick a single optimal chromosome from the final population, we selected a solution from the first Pareto front with well-balanced results for all three modes. The chosen sensor arrangement is illustrated in Figure 7a. As can be observed, most of the measurement points are arranged near the fixed end, where there is a higher strain concentration. Additionally, note that the obtained distribution is slightly asymmetric. This issue could be easily addressed by introducing constraints in the optimization process. However, in the present study we decided to follow a more general approach. In Figure 7b, a uniformly distributed sensor pattern is also shown, intended for validating the optimization process. Such pattern will be utilized for SEA pre-extrapolation and, subsequently, the iFEM will be applied; the results obtained will then be compared with those coming from the sensor layout generated through optimization.

**Figure 8:**
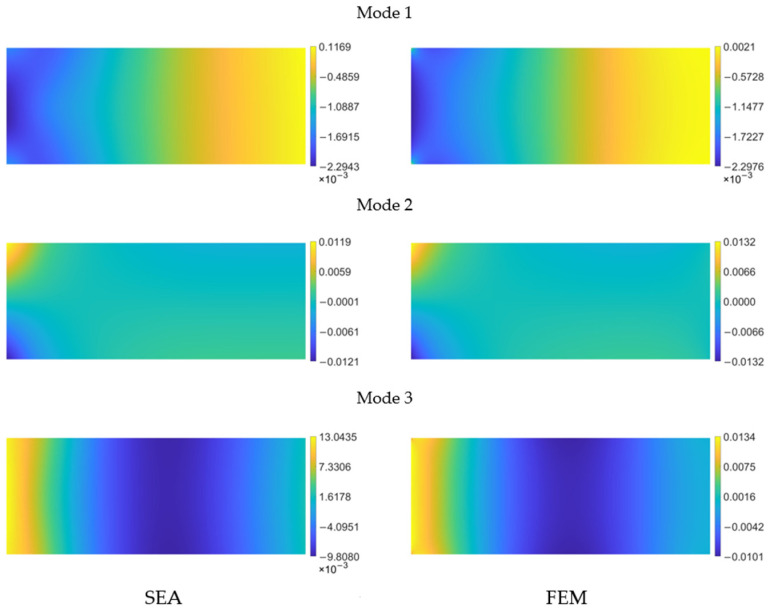
Pre-extrapolated $k_{xx}\;\left(\frac{1}{m}\right)\;$ on the left vs. reference on the right for the first 3 modes of the cantilevered plate.

By applying the smoothing element analysis with the optimal sensor arrangement, we are able to generate the continuous spatial distribution of the three curvature components $k_{xx}$ , $k_{yy}$ , and $k_{xy}$ for each mode. In Figure 8, the curvature distribution of $k_{xx}$ (in 1/m ) pre-extrapolated with the pattern from Figure 7a is shown as an example and is compared to the reference field provided by the finite element model.

As can be observed, the reconstructed distributions visually appear very similar to their reference counterpart, with nearly coincident scale values. A similar behavior is exhibited by the other two curvature components. Overall, the correlation for $k_{yy}$ tends to be slightly lower for each mode, given that it is the smallest among the components. Consequently, its reconstruction is less accurate, though its influence on the displacement field is also minor. The smoothed field obtained through the SEA is then employed as input for the inverse finite element method utilizing the fine mesh shown on the left in Figure 6.

**Figure 9:**
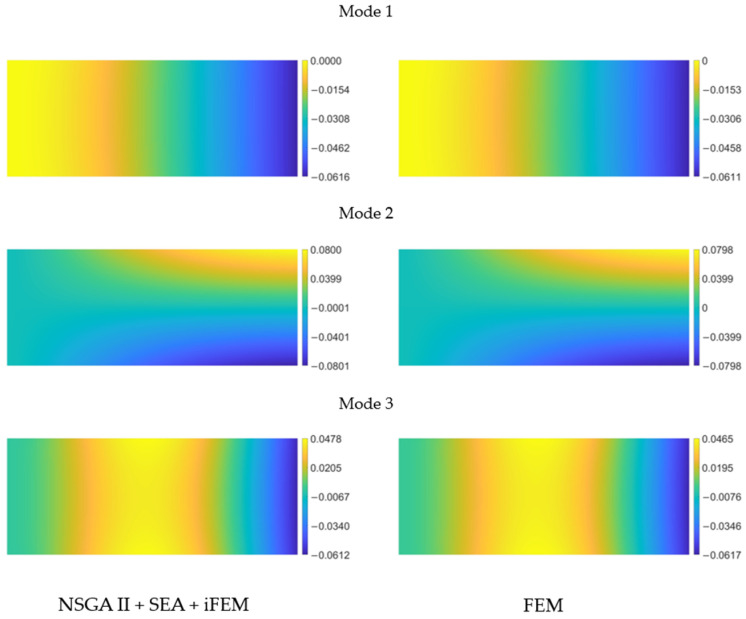
Cantilevered plate’s first three modes deflection $w\;\left(m\right)$ for iFEM aided by NSGA-II and SEA (**left**) and reference solution (**right**).

For the three mode shapes under consideration, the only displacement field component different from zero is w (since u=v=0 ). In Figure 9, a comparison is shown between the deflection reconstructed by the iFEM aided by optimization and pre-extrapolation (on the left), and the reference solution from the FE model (on the right). The results are presented in dimensional form ( m ). By analyzing the graphs, it appears evident that the selected sensor layout effectively captures the trends and values of the w field.

**Figure 10:**
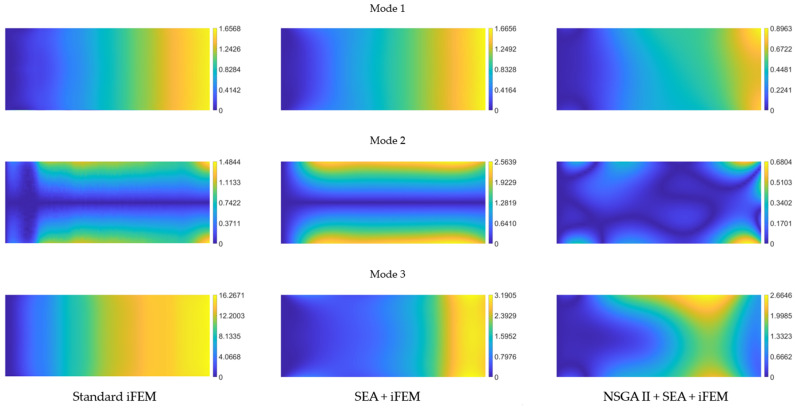
Cantilevered plate’s reconstruction percentage error of the first three modes for standard iFEM (**left**), smoothed iFEM with uniformly distributed sensors (**center**), and smoothed iFEM with optimal layout (**right**).

To better quantify the method’s accuracy, we define the reconstruction error as follows:

(22) $$err\left(x\right)=\frac{\left|w^{rec}\left(x\right)-w^{ref}\left(x\right)\right|}{max\left(\left|w^{ref}\left(x\right)\right|\right)}\cdot 100$$

where $w^{rec}$ is the reconstructed deflection and $w^{ref}$ is the reference FE one. This metric allows us to compare the shape-sensing capabilities of the iFEM, the iFEM with pre-extrapolation using the pattern in Figure 7b, and the iFEM with pre-extrapolation using the optimized pattern in Figure 7a. The error percentage for each of these three approaches is presented in Figure 10. As evident from the images on the left, the standard iFEM accurately captures the first two modes with minimal errors (below 2% in both cases). However, it fails in reconstructing appropriately the third mode, with a maximum error of 16%. In the center of Figure 10, the reconstruction error using the pattern from Figure 7b is depicted. Notably, this sensor layout shows no improvement over the standard iFEM for the first mode, and it even worsens the results for the second one (increasing the maximum error and displaying a broader error distribution across the domain). However, there is a substantial increase in accuracy for the third mode, with improvements exceeding 10% on the maximum error. On the right, the error trends obtained using the optimized pattern from Figure 7a are shown. This layout yields lower error values compared to the other two approaches (maximum values below 1% for the first two modes and around 2.6% for the third), with more confined error distributions in space. This comparison highlights the significant benefits, in terms of overall accuracy, when employing pre-extrapolation coupled with optimal sensor placement compared to conventional iFEM and smoothed iFEM with a uniformly distributed sensor layout.

### 3.2. Clamped Square Plate

**Figure 11:**
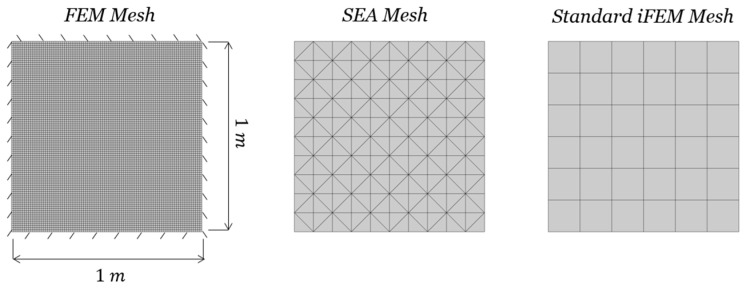
Clamped square plate. FE mesh (**left**); SEA mesh (**center**); standard iFEM mesh (**right**).

The second case study considered is a square plate clamped on all sides, as shown in Figure 11. The plate has dimensions of 1 m in length, 1 m in width, and a thickness of 0.01 m . The geometric constraints enforcing u=v=w=0  and $\theta_{x}=\theta_{y}=\theta_{z}=0$ are applied on each side. The structure is assumed to be made of an isotropic material, with properties similar to an aluminum alloy ( E=70 Gpa , ν=0.3 , $\rho =2700\;\mathrm{kg}/m^{3}$ ). The plate was modeled using MSC NASTRAN, with a mesh comprising 90 × 90 CQUAD4 elements, which was selected after a convergence study, as illustrated on the left in Figure 11. Employing the SOL 103 solver, modal analysis was conducted to extract the structure’s first four natural modes, identified at frequencies of 88.12 Hz , 179.57 Hz , 179.57 Hz , and 264.40 Hz . As conducted in the previous case study, the mesh shown on the left of Figure 11 is also employed by the smoothed iFEM. In this study, we employ 36 strain rosettes to monitor the four mode shapes. In the sensor positioning optimization, we make use of a triangular SEA mesh of 200 elements, depicted in the middle of Figure 11. Similar considerations apply to the SEA mesh dimensions as those made for the clamped rectangular plate. The final mesh, represented on the right in Figure 11, corresponds to the standard iFEM one and consists of 6 × 6 inverse elements, each featuring a strain rosette at its centroid.

**Figure 12:**
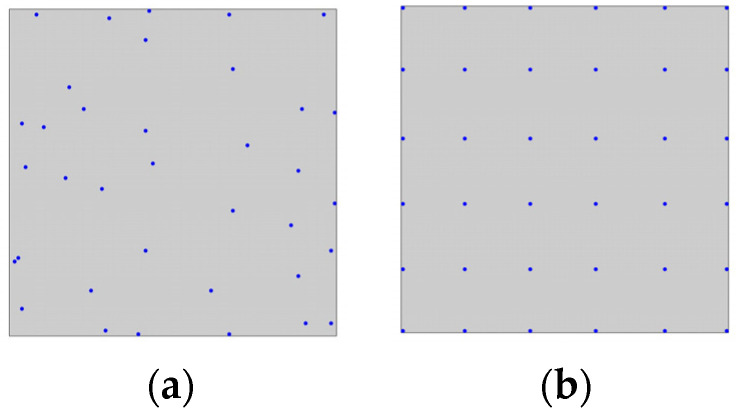
(**a**) Selected optimal sensor arrangement for the clamped square plate; (**b**) uniform sensor placement used for validation.

Within the genetic algorithm, a population of 300 chromosomes was employed, and the maximum number of iterations was set at 150. The crossover and mutation probability are set at $p_{cross}=0.9$ and $p_{mut}=0.1$ , respectively; the maximum number of mutated genes is selected as $n_{mut}^{max}=3$ ; and the number of elites is equal to $N_{pop}$ . In the calculation of the fitness function, a regularly spaced grid of 289 sampling points was utilized. In this case study as well, the optimization process was repeated multiple times, and the optimal individual for comparison with the standard iFEM was chosen from the first Pareto front among those displaying balanced results for each of the four modes. The selected pattern is illustrated in Figure 12a. On the other hand, a uniformly distributed sensor pattern used for validating the optimization process is displayed in Figure 12b.

**Figure 13:**
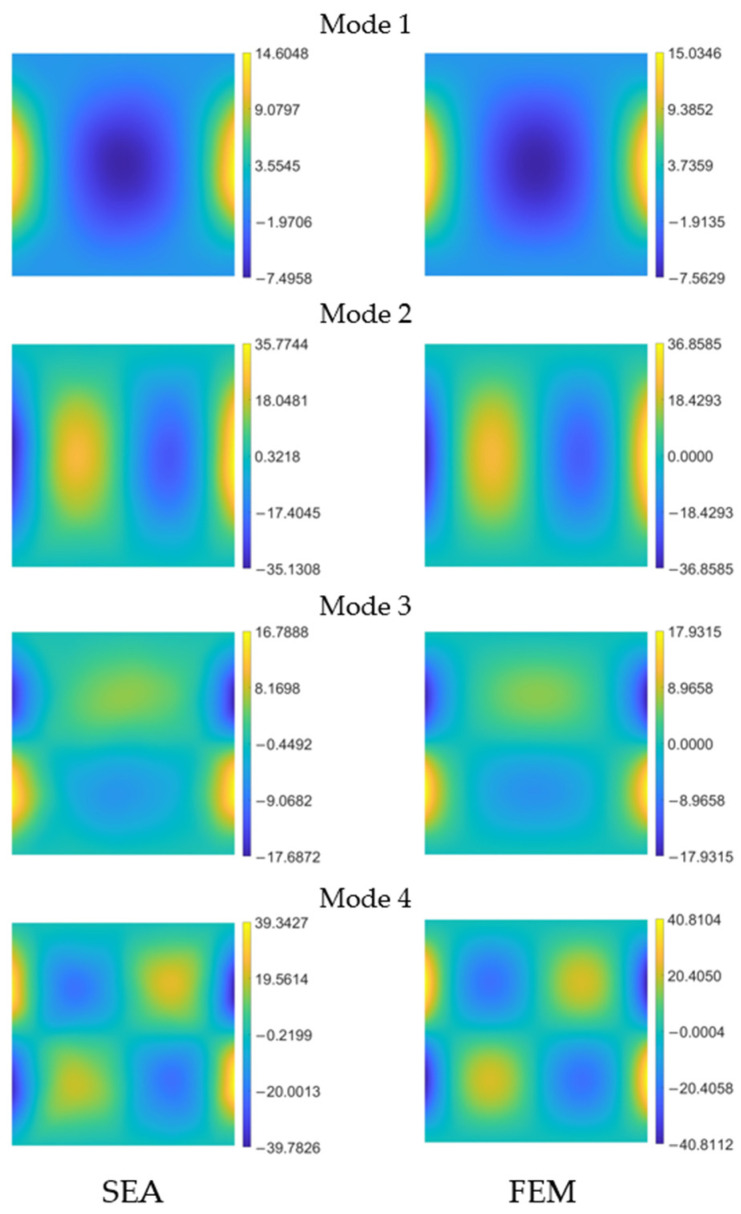
Pre-extrapolated $k_{xx}\;\left(\frac{1}{m}\right)\;$ on the left vs. reference on the right for the first four modes of the clamped plate.

In Figure 13, the SEA $k_{xx}$ ( 1/m ) curvature obtained using the optimal sensor pattern in Figure 12a is compared with the FEM reference for each mode.

The shape of the field is accurately captured and, numerically, the values are closely aligned. Similar behaviors are observed for the other two curvature components, $k_{yy}$ and $k_{xy}$ , and are not reported for brevity. Since the sensor pattern is not symmetrical, asymmetry is evident in the pre-extrapolated curvatures, subsequently affecting the reconstructed displacement field. However, it should again be noted that adopting simple strategies can easily rectify such asymmetry.

**Figure 14:**
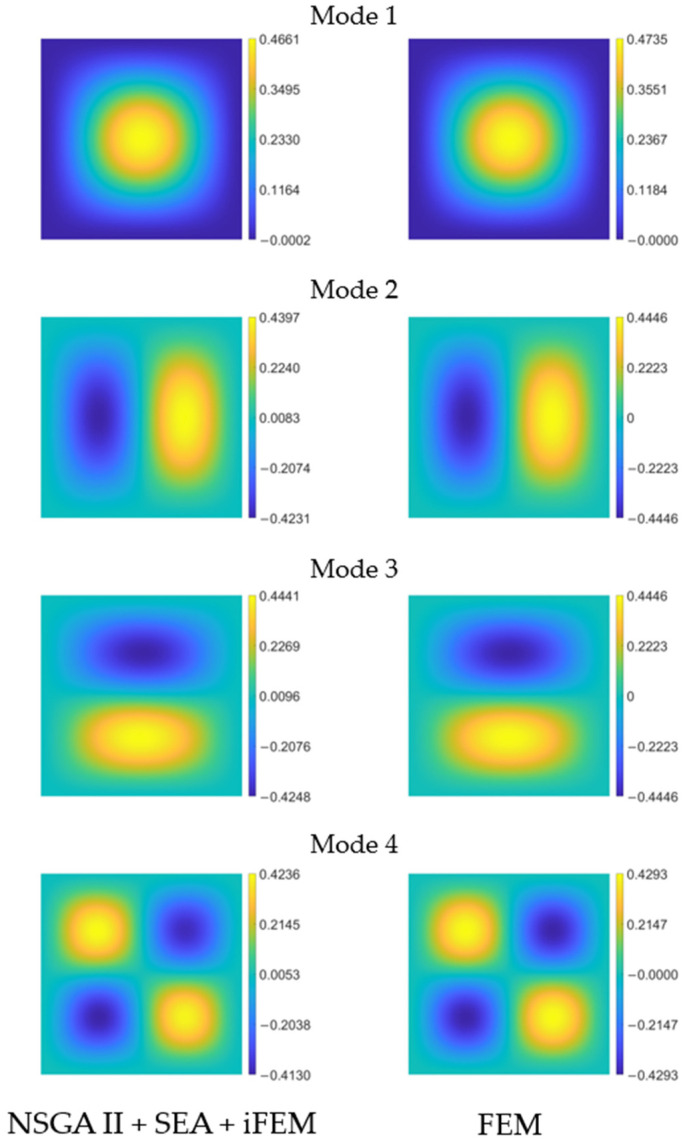
Clamped plate’s first four modes deflection $w\;\left(m\right)$ for iFEM aided by NSGA-II and SEA (**left**) and reference solution (**right**).

For the four mode shapes under consideration, the only displacement field component different from zero is w (since u=v=0 ). In Figure 14, a comparison is shown between the deflection (in m ) reconstructed by the iFEM aided by optimization and pre-extrapolation (on the left), and the reference solution from the FE model (on the right). By analyzing the graphs, one can observe that also in this case study the optimal sensor layout can reconstruct the spatial distribution of w appropriately.

**Figure 15:**
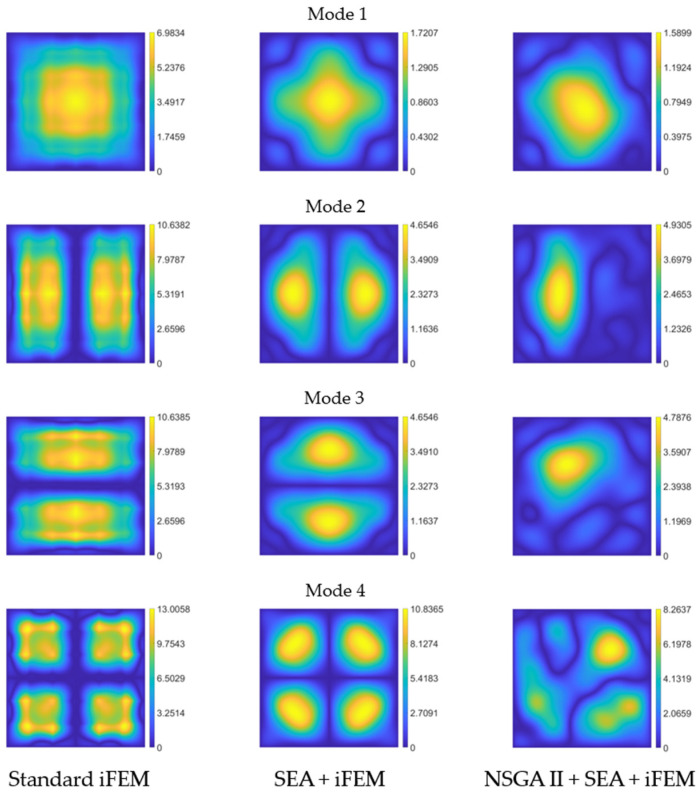
Clamped plate’s reconstruction percentage error of the first four modes for standard iFEM (**left**), smoothed iFEM with uniformly distributed sensors (**center**), and smoothed iFEM with optimal layout (**right**).

We use the error defined in Equation (22) to draw a comparison between the shape-sensing performance among the standard iFEM, the iFEM with pre-extrapolation using the pattern in Figure 12b, and the iFEM with pre-extrapolation using the optimized layout in Figure 12a. The percentage error for each of these approaches is illustrated in Figure 15. As observed from the color maps on the left, the application of the standard iFEM to the clamped plate results in significant errors: the maximum error is approximately 7% for the first mode, around 10% for the second and third modes, and approximately 13% for the fourth mode. Conversely, the central graphs in Figure 15 demonstrate the beneficial impact of using pre-extrapolation with a uniformly distributed sensor layout, leading to a substantial reduction in error compared to the conventional iFEM: the maximum error for the first mode is 1.72%, for the second and third modes is 4.63%, and for the fourth mode is 10.83%. The graphs on the right represent the error obtained with the sensor pattern resulting from the optimization process. While the maximum error for the first three modes remains close to that achieved with uniformly distributed sensors (slightly lower for the first mode and slightly higher for the second and third modes), an analysis of the spatial distribution of the error reveals that, for modes 2 and 3, the maximum error is confined to one of the two peaks, with the rest of the field exhibiting errors below 2%. This implies that, although the maximum error is of the same order as the uniformly distributed layout, the use of the pattern in Figure 12a leads to a reduction in the average error. For the fourth and final mode, there is a 2.5% improvement in the maximum error compared to the uniformly distributed pattern, with the error once again primarily localized to one of the four peaks, while the rest of the geometry maintains a sufficiently low error. Overall, for this case study, the use of pre-extrapolation yields substantially better results than the standard iFEM, and opting for an optimized pattern over a uniformly distributed one for SEA generally lowers the average error in the field.

## 4. Conclusions

This study introduces an innovative approach to the shape-sensing problem involving plate structures by integrating smoothed element analysis (SEA) and the non-dominated sorting genetic algorithm II (NSGA-II) with the inverse finite element (iFEM) methodology. The proposed method optimizes sensor placement to pre-extrapolate strain fields with minimal root mean square error compared to reference solutions across diverse loading conditions. The pre-extrapolated strains are then utilized as input for the inverse finite element method. The effectiveness of this approach is demonstrated through notable improvements over the standard iFEM in reconstructing a set of mode shapes in two case studies involving a cantilevered rectangular plate and a clamped square plate.

This methodology effectively enhances monitoring capabilities with a limited number of strain sensors and is not necessarily confined to planar problems. The SEA has demonstrated successful application to three-dimensional structures like curved shells [45] and stiffened panels [46]. Therefore, the integration of sensor placement optimization with strain pre-extrapolation in the implementation of the inverse finite element method can be extended to industrial systems, offering a practical and efficient choice for structural health monitoring (SHM) applications.

## Data Availability

The data are contained within the article.
